# Characterizing the comfort limits of forces applied to the shoulders, thigh and shank to inform exosuit design

**DOI:** 10.1371/journal.pone.0228536

**Published:** 2020-02-12

**Authors:** Matthew B. Yandell, David M. Ziemnicki, Kirsty A. McDonald, Karl E. Zelik

**Affiliations:** 1 Dept. of Mechanical Engineering, Vanderbilt University, Nashville, TN, United States of America; 2 Dept. of Physical Medicine & Rehabilitation, Vanderbilt University, Nashville, TN, United States of America; 3 Dept. of Biomedical Engineering, Vanderbilt University, Nashville, TN, United States of America; National University of Singapore, SINGAPORE

## Abstract

Recent literature emphasizes the importance of comfort in the design of exosuits and other assistive devices that physically augment humans; however, there is little quantitative data to aid designers in determining what level of force makes users uncomfortable. To help close this knowledge gap, we characterized human comfort limits when applying forces to the shoulders, thigh and shank. Our objectives were: (i) characterize the comfort limits for multiple healthy participants, (ii) characterize comfort limits across days, and (iii) determine if comfort limits change when forces are applied at higher vs. lower rates. We performed an experiment (*N* = 10) to quantify maximum tolerable force pulling down on the shoulders, and axially along the thigh and shank; we termed this force the comfort limit. We applied a series of forces of increasing magnitude, using a robotic actuator, to soft sleeves around their thigh and shank, and to a harness on their shoulders. Participants were instructed to press an off-switch, immediately removing the force, when they felt uncomfortable such that they did not want to feel a higher level of force. On average, participants exhibited comfort limits of ~0.9–1.3 times body weight on each segment: 621±245 N (shoulders), 867±296 N (thigh), 702±220 N (shank), which were above force levels applied by exosuits in prior literature. However, individual participant comfort limits varied greatly (~250–1200 N). Average comfort limits increased over multiple days (*p*<3e-5), as users habituated, from ~550–700 N on the first day to ~650–950 N on the fourth. Specifically, comfort limits increased 20%, 35% and 22% for the shoulders, thigh and shank, respectively. Finally, participants generally tolerated higher force when it was applied more rapidly. These results provide initial benchmarks for exosuit designers and end-users, and pave the way for exploring comfort limits over larger time scales, within larger samples and in different populations.

## Introduction

Exosuits (soft exoskeletons) are wearable devices made primarily from soft/flexible structures that physically augment, assist, or enhance human movement or posture. Exosuits offer exciting potential to enhance human health and performance [1–5] with both passive and powered implementations, and applications in medical, military, and industrial domains. However, one of the most challenging aspects of exosuit design–as well as design for many other wearable assistive devices, from prostheses to rigid exoskeletons–involves the physical interfaces that connect the device to the person [1], [6–8]. Each physical interface consists of synthetic materials (e.g., straps, cuffs, sleeves) that are part of the exosuit, as well as the underlying biological tissues (e.g., skin, fat, muscle and other tissues [9], [10]). When exosuit forces are applied to the user, the synthetic and biological interface components can physically displace, deform, and/or shift relative to each other. These interface dynamics affect how force and power are transmitted to the user [7]. Higher forces can provide more assistance, but also subjectively affect user comfort and experience. If applied forces are too high, users may become too uncomfortable to continue using the device. Here we refer to the maximum force which users tolerate as their *comfort limit* (formally defined in Methods).

For existing and future exosuits, it is important to determine what magnitudes of force can be applied to physical interfaces on various body segments before user comfort limits are reached. User comfort is commonly stated in prior literature to be an important aspect of exosuit and exoskeleton design [1], [8]. However, most studies either mention this issue based on anecdotal feedback from their participants, or report the results of subjective surveys using Likert, visual analog or other qualitative self-report scales (e.g.,[11], [12]). There is little quantitative data and a lack of published guidelines on comfort limits for wearable devices. In general, it is not known how much force on a given body segment leads to discomfort, or how the comfort limit varies across individuals, loading rates, or time scales. Understanding these practical limits for applying forces can help provide design guidelines for the development of new exosuits, or inform the refinement of springs, actuators, or controllers in existing devices to maximize assistance without exceeding user comfort limits.

A first step towards addressing this large knowledge gap is to quantify comfort limits over short time scales (e.g., seconds), to gain insight into the magnitude, range and variability of user comfort limits. This information will inform exosuit device design and longer-term testing (e.g., over minutes, hours or days). The purpose of this study was to quantify these comfort limits for exosuit interfaces when pulling forces were applied to three key segments of the body (shoulders, thigh and shank), which are commonly used in exosuit designs (e.g., [1], [3–5]). Our specific objectives were three-fold: (i) characterize the comfort limits for multiple healthy participants, (ii) characterize comfort limits across days, and (iii) determine if comfort limits change when forces are applied at higher vs. lower rates.

## Methods

### Summary

**Fig 1:**
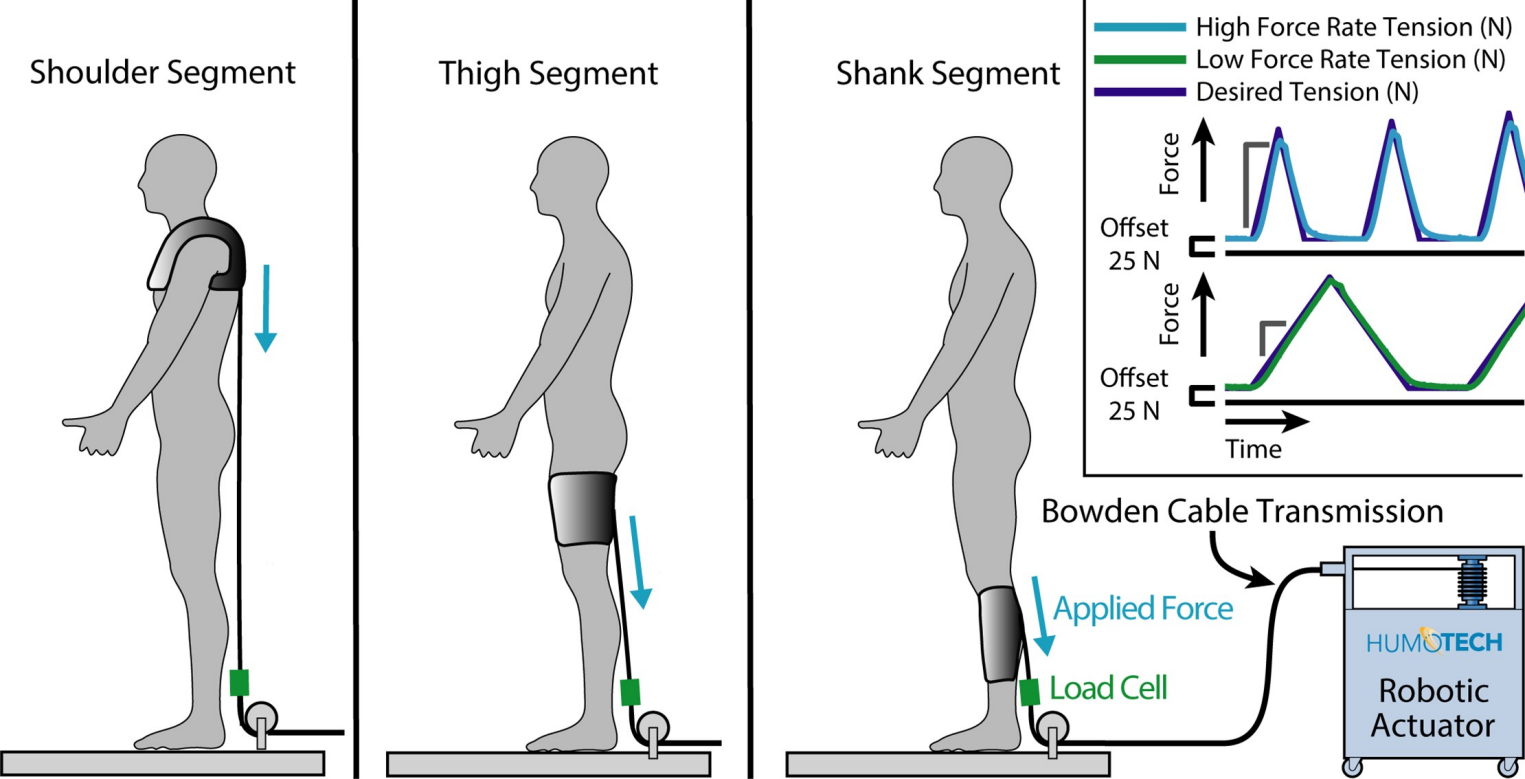
Experimental setup. A robotic actuator was used to apply pulling forces to three separate interface locations. The subject stabilized themselves in a custom scaffold (not depicted for clarity; see Appendix). Inset: Desired tension force (blue) vs. actual tension force (green). The peak force of the triangle profile increased incrementally by 50 N from the beginning value. The triangle wave force profile was determined by the specified loading rate. The initial peak force value (peak value of the first triangle) and the rate of force application (high vs. low) were randomized for each segment, subject and session. For the shoulders, the high force rate was 300 Ns^-1^ and the low force rate was 100 Ns^-1^; for the shank and thigh, the high force rate was 3000 Ns^-1^ and the low force rate was 1000 Ns^-1^.

For the shoulders, thigh, and shank, we quantified the maximum pulling force that could be applied before participants reported that they had reached their *comfort limit* (formally defined below). We fabricated exosuit interfaces for the shoulders, thigh, and shank, and systematically applied forces using a robotic actuator. The actuator generated a series of pulling forces (Fig 1) that increased in peak magnitude every cycle. Subjects then used a push-button off-switch to stop the actuation system and remove the applied force when they did not want to feel a higher level of force (see specific instructions to subjects below). The experimental protocol was designed to address the three specific objectives stated above.

### Experimental setup

We used an offboard robotic actuator (Humotech, Pittsburgh, USA) to apply pulling forces to exosuit interfaces worn by the subjects, while continuous force data were recorded at 1000 Hz by an inline single-axis load cell (LCM300, Futek, Irvine, USA). Subjects stabilized themselves in a custom-built scaffold (depicted in Appendix) as the actuator force was applied vertically downward to the interfaces located on the shoulders, thigh, and shank (Fig 1). Given the participants’ body postures, the pulling directions were not perfectly aligned along the axial directions of the body segments. The pulling direction was roughly 5–10 degrees off the longitudinal axis of the shank, roughly 30–35 degrees off the longitudinal axis of the thigh, and within about 5 degrees of the longitudinal axis of the trunk when pulling on the shoulders (Fig 1).

Subjects also completed short surveys at the beginning and end of each session to verify that the testing did not cause persistent discomfort or soreness. Between conditions, subjects were given short breaks (typically less than 30 seconds) or longer breaks upon request. All human subject studies were approved by the Vanderbilt University Institutional Review Board (IRB) and the subjects gave informed written consent prior to participation. The individual in this manuscript (depicted in the Appendix) has given written informed consent (as outlined in PLOS consent form) to publish these images and case details.

### Subjects and conditions

We tested healthy adult subjects (*N* = 10; 5 male, 5 female; Age: 24 ± 2 years old; Height: 1.8 ± 0.1 m; Mass: 69.3 ± 12.1 kg) across four sessions, which were separated by at least one full day and at most 18 days (5.5 ± 4.3 days). Each subject was given a written set of instructions for participating in the study (see Instructions to Subjects). For each session, we tested interfaces on three different locations on the body (shoulders, thigh and shank). These segments are commonly used by exosuits to apply assistive forces to the user [1], [3–5]. During each session, the subject donned an exosuit interface at a given body location: a thermoplastic elastomer thigh or shank sleeve with nylon wrap attached via hook-and-loop, or a shoulder harness (see Appendix for more interface details). We had multiple sizes of interface, and selected the size that best fit each user’s shank and thigh (see Appendix). For all 10 subjects, we tested comfort limits on the right side thigh and shank segments for all four testing days (see Appendix). We tested only the right side based on our expectation that participants would generally be symmetrical in their sensations of discomfort across limbs; though future experiments could be designed to assess this expectation. We used one shoulder harness that was adjusted to fit each participant. Each interface was then connected to the offboard actuator, which applied incrementally increasing downward pulling forces in a triangle wave (Fig 1). When the subject felt uncomfortable to the point that they did not want to feel a higher level of force, they pressed the off-switch that immediately disabled the actuator and removed the applied force. Alternatively, if the peak forces reached 1200 N, the maximum approved force from our IRB protocol, then the trial was stopped by the experimenter.

Three body locations (shoulders, thigh and shank) were independently loaded ten times–five trials at a low loading rate and five trials at a high rate–for a total of 30 trials per subject per session. For the shoulders, the high force rate was 300 Ns^-1^ and the low force rate was 100 Ns^-1^; for the shank and thigh, the high force rate was 3000 Ns^-1^ and the low force rate was 1000 Ns^-1^. The applied force rates were chosen based on the typical ranges reported in prior exosuit literature [5], [7], [13–16]. The magnitude of the first peak and the applied force rate (high vs. low) was randomized for each segment, test session, and subject in order to blind the subjects to conditions and discourage gamification (e.g., to prevent users from being able to simply count the number of loading cycles to figure out which peak force they had reached). Ten subjects completed the protocol. However, three subjects only completed the first two days of shank testing, as they reported some transient discomfort on the third testing day. Thus we erred on the side of not collecting additional shank data for these three subjects, and they were excluded from statistical comparisons on shank comfort limits (but were included in the thigh and shoulder analysis). To mitigate sweating, which can cause slippage of the interface, subjects applied antiperspirant to their shank and thigh segments the night before testing, and a fan blew air over the subject’s leg to keep them cool during the experiment. In some cases there was minor slippage of the shank and thigh interfaces down the leg (15 mm or less) over the course of multiple trials. If there was substantial slippage (as noticed by the experimenter or the subject), then the interface was removed, the liner and subject’s skin dried via a fan, and the interface was reset to the original location prior to continuing testing.

### Instructions to subjects

To ensure uniformity of instructions to subjects, we provided the following set of written instructions to each participant during each session. Any questions the subjects had were then answered prior to the experimental session.

“*Exosuits are devices that attach to the body and physically assist movement. Exosuits are being developed to improve the quality of life for people with disabilities, to augment the strength of individuals performing strenuous jobs, and to help keep workers safe and injury-free. We are interested in understanding how much force an exosuit can apply to various parts of the body before this force becomes uncomfortable. For each trial, please press the off-switch when you feel uncomfortable to the point that you would not want to feel a higher level of force. Please ask the experimenter any questions you may have.*”

### Definition of comfort limit

Here we defined the comfort limit as the maximum force experienced by the subject during a trial. This limit corresponded to either (i) the maximum force prior to the subject pressing the off-switch or (ii) the peak force at the very end of the trial, in which case the maximum approved force under our current IRB protocol (1200 N) was reached.

### Data processing

Forces were low-pass filtered by a third-order Butterworth filter (cutoff frequency of 10 Hz), and used to determine the comfort limit in each trial. Across trials for each individual subject, the median comfort limit was computed. The median was used rather than the mean because it is less sensitive to the ceiling effect imposed by the maximum approved force, i.e., less likely to be skewed by the subset of trials that reached the maximum approved force limit. The subject-specific median comfort limits were then used to compute group averages on a segment-by-segment basis as detailed below.

**Fig 2:**
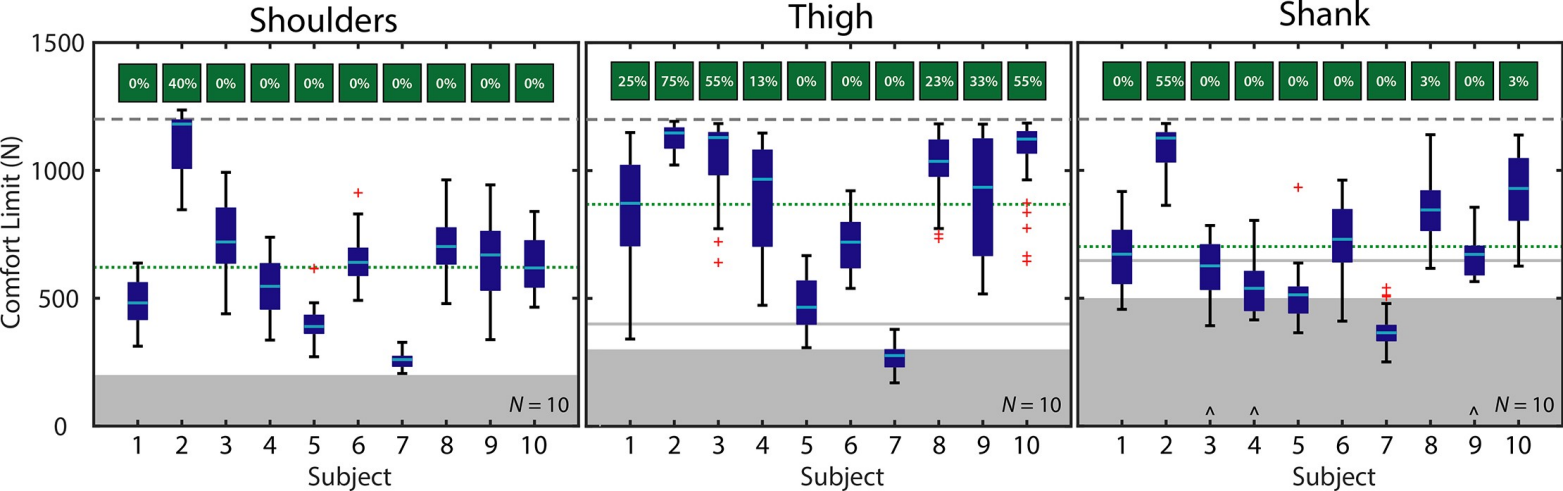
Inter-subject comfort limits. Boxplots depict individual subject results. Boxplots indicate subject median (cyan line), 25th and 75th quartiles (dark blue box), whiskers (extending to the farthest data point or 1.5x the interquartile range, whichever is nearer the median) and outliers (red cross markings). Three subjects (denoted with ^) only completed the first two sessions of testing on the shank. Percentage of trials that reached the maximum approved force limit are depicted in green boxes above each column. The 1200 N maximum approved force limit is denoted with a dashed gray line. The group mean comfort limit is denoted with a dotted green line. The gray boxes indicate the approximate range of forces applied by exosuits in prior literature during dynamic tasks [1], [2], [14–16]; the gray solid lines indicate maximum peak forces applied by individual studies during static tasks [4], [13].

To characterize inter-subject differences (Fig 2), the median comfort limit was computed from all data for each subject (40 trials per body segment). This yielded one comfort limit per subject, or 10 in total. Next, the group mean and standard deviation was computed from these 10 values.

**Fig 3:**
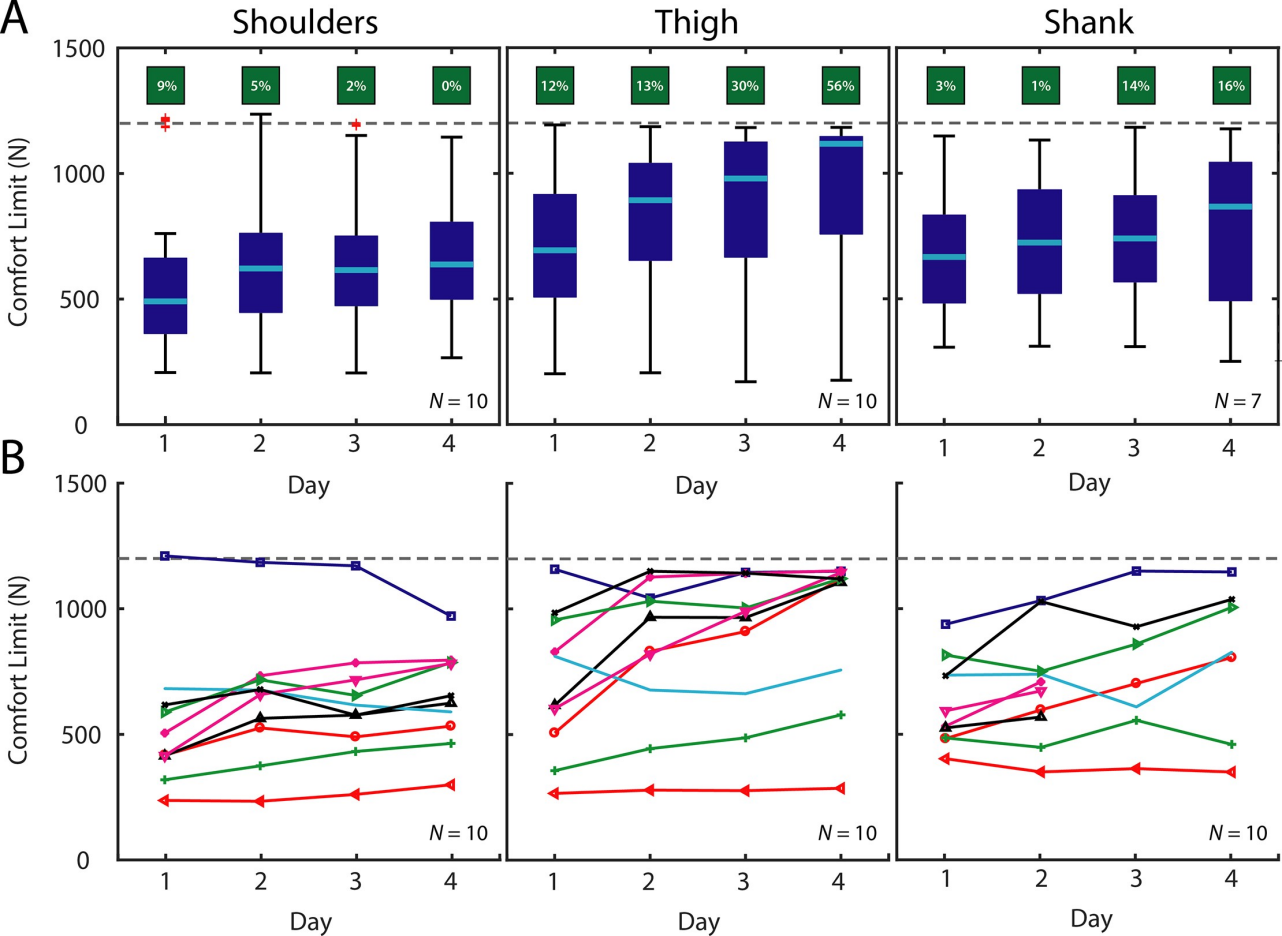
Inter-Day comfort limits. A) Group results. Boxplots depict group results for each testing day. Percentage of trials that reached the maximum approved force limit are depicted in green boxes above each column. B) Subject-specific results. Each marker (colored shape) depicts the median comfort limit for a single subject across multiple days. The 1200 N maximum approved force limit is denoted with a dashed gray line.

To characterize inter-day differences, (Fig 3), the median comfort limit was computed from all data for each day for each subject (10 trials per segment). Then, the group mean and standard deviation was computed across subjects for each day, yielding one mean and one standard deviation for each of the four days. Separately, Friedman’s test (the non-parametric alternative to a repeated measures ANOVA) was used to assess if there were differences in comfort limit due to testing day, using combined raw data points from all subjects to compare between days (alpha = 0.05). Wilcoxon signed rank tests (alpha = 0.05, with Holm-Bonferroni correction for multiple comparisons) were used to compare between individual days.

**Fig 4:**
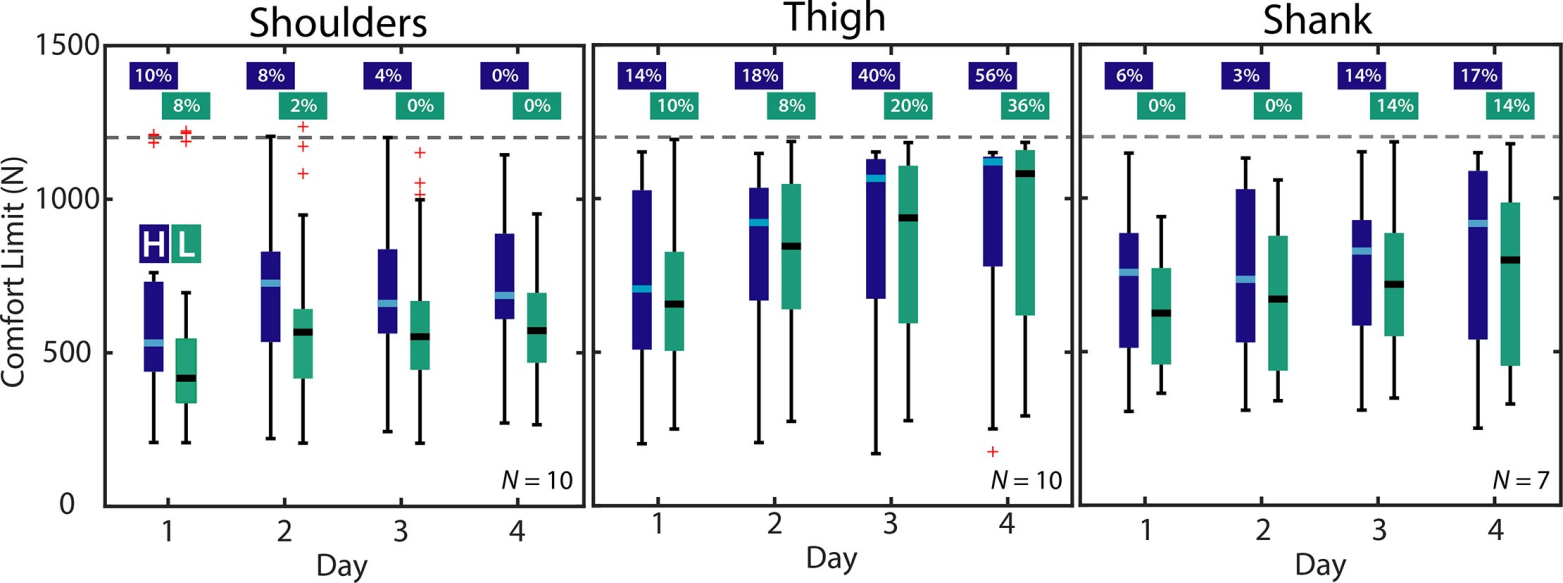
Effect of force rate on comfort limits. Boxplots depict group results for each force rate across days. High force rate results are in dark blue (“H”), and low force rates are depicted in light green (“L”). Percentage of trials that reached the maximum approved force limit are depicted above each column. The 1200 N maximum approved force is denoted with a dashed gray line.

To assess force rate differences, (Fig 4), the median comfort limit was computed from all data for each day, at each rate, for each subject (5 high force rate trials and 5 low force rate trials per segment). Then the group mean and standard deviation was computed across subjects for each day at each rate. On each day, we computed the percentage change in comfort limit due to force rate using the difference in group means at the two rates. These percent changes were then averaged across days to compute the mean percentage change. Separately, the Wilcoxon signed rank test (alpha = 0.05) was used to assess the effect of force rate, using combined raw data points from all subjects at each rate to compare between rates for each day.

## Results

### Inter-subject

#### Shoulders

The comfort limit on the shoulders had a group mean of 621 ± 245 N. The lowest individual subject median was 260 N, while the highest was 1181 N. Only one subject (Subject 2, Fig 2) reached the maximum approved force limit of 1200 N on individual trials.

#### Thigh

The comfort limit on the thigh segment had a group mean of 867 ± 296 N. The lowest individual subject median was 276 N, while the highest was 1147 N. Seven subjects (Subjects 1–4 and 8–10, Fig 2) reached the maximum approved force limit on individual trials.

#### Shank

The comfort limit on the shank segment had a group mean of 702 ± 220 N. The lowest individual subject median was 365 N, while the highest was 1126 N. Three subjects (Subjects 2, 8, and 10, Fig 2) reached the maximum approved force limit on individual trials. The three subjects who only completed the first two days of testing are included in Fig 2 and in the group mean comfort limit reported above. See Appendix for a detailed Table of subject-specific results.

### Inter-Day

#### Shoulders

The comfort limit on the shoulders increased from a group mean of 540 ± 272 N on Day 1 up to 649 ± 193 N on Day 4 (Fig 3), a 20% increase. We did observe an effect on comfort limit due to testing day (*p* < 3e-09). Additionally, The Day 1 comfort limit was different from Days 2, 3, and 4 (*p* < 2e-06). Based on visual inspection of subject-specific data, 7 subjects’ comfort limits trended up over time, 2 trended downward, and 1 remained roughly neutral (Fig 3).

#### Thigh

The comfort limit on the thigh segment increased from a group mean of 709 ± 288 N on Day 1 to 954 ± 307 N on Day 4 (Fig 3), a 35% increase. We did observe an effect on comfort limit due to testing day (*p* < 6e-20). Each Day’s comfort limit was different than every other Day (*p* < 2e-05). Based on visual inspection of subject-specific data, 7 subjects’ comfort limits trended up over time, none trended downward, and 3 remained roughly neutral (Fig 3).

#### Shank

The comfort limit on the shank segment increased from a group mean of 658 ± 200 N on Day 1 to 806 ± 299 N on Day 4 (Fig 3, *N* = 7), a 22% increase. We did observe an effect on comfort limit due to testing day (*p* < 3e-5). Each Day’s comfort limit was different than every other Day (*p* < 0.014), except when comparing Day 1 to Day 2. Based on visual inspection of subject-specific data, 8 subjects’ comfort limits trended up over time, 1 subject trended downward, and 1 remained roughly neutral (Fig 3). See Appendix for a detailed Table of results.

### Force rate

#### Shoulders

When the actuator pulled at a higher vs. lower rate (300 Ns^-1^ vs. 100 Ns^-1^), then comfort limit was 22% higher (mean percentage change across days); 667 N vs. 554 N (Fig 4). The comfort limit was higher for the shoulders at the higher loading rate for each Day (*p* < 4e-08).

#### Thigh

When the actuator pulled at a higher vs. lower rate (3000 Ns^-1^ vs. 1000 Ns^-1^), then the comfort limit was 4% higher (mean percentage change); 861 N vs. 831 N (Fig 4). The comfort limit difference was higher at the higher loading rate for the thigh only on Day 3 (*p* < 0.013); however, it is worth noting that this statistical comparison was likely affected by a considerable number of trials reaching the maximum approved force limit. Also of note, a higher percentage of high loading rate trials reached the maximum approved force than low loading rate trials (Fig 4).

#### Shank

When the actuator pulled at a higher vs. lower rate (3000 Ns^-1^ vs. 1000 Ns^-1^), then the comfort limit was 10% higher (mean percentage change); 769 N vs. 702 N (Fig 4, *N* = 7). The comfort limit was higher at the higher loading rate for the shank on Days 1, 2, and 4 (*p* < 0.002), but not different due to loading rate on Day 3. See Appendix for a detailed table of results.

## Discussion

To inform exosuit design, we characterized the pulling force comfort limits on various body segments. We found that, on average, our healthy participants exhibited comfort limits of about 620–870 N (≈ 0.9–1.3 times the average participant body weight, Fig 2) on each body segment. However, individual subjects and segments varied greatly, ranging from about 250–1200 N (≈ 0.4–1.7 body weights, Fig 2). We found that the comfort limit tended to increase over multiple days of testing for most subjects; the group mean comfort limits increased from about 550–700 N on Day 1 to about 650–950 N on Day 4 (≈ 1–1.4 times participant body weight, Fig 3). Also, we observed that users could generally tolerate higher force when it was applied more rapidly (Fig 4). This study fills an important knowledge gap relevant to exosuit designers and users. These comfort limits may help inform actuator and transmission design requirements, and/or provide insight on whether a specified amount of exosuit assistance is likely to cause discomfort, in which case a designer may be able to modify their design approach even before fabrication and prototyping. We note that there is no consensus on how comfort (or comfort limits) are measured experimentally, particularly for exoskeletons/exosuits. Our comfort limit definition reflects the key information we desired to understand as device designers. There are a variety of alternative or complementary ways to assess comfort and limits of comfort (e.g., [12], [17], [18]).

In general, the comfort limits observed were higher than the forces applied by exosuits in prior literature. Exosuits that pull on the thigh have reported force magnitudes of about 150–300 N (with one example of 400 N in a static task); exosuits that pull on the shank have reported force magnitudes of about 150–500 N (with one example of 650 N in a static task) [1], [2], [4], [13–16], [19]. However, in this study the average comfort limits we found were >700 N (>1 body weight, green dotted lines in Fig 2) for the thigh and shank. Likewise, for back-assist exosuits that pull downward on the shoulders, force magnitudes in prior literature have been on the order of about 50–200 N [5], [20], which is considerably lower than the group mean comfort limit in this study of >600 N (Fig 2). Indeed, all subjects in this study had comfort limits on the shoulders above the applied exosuit force magnitudes reported in literature [5], [20].

Nevertheless, there was high inter-subject variability for all segments (Fig 2). The reason for the high inter-subject variability is currently unknown, though this high variability in comfort perception is also seen in algometry studies [21], [22]. It may be due to innate physiological differences, or differences in how individuals experience discomfort. It is also conceivable that the interfaces used did not fit certain subjects as well as other subjects, and that, through improved interface design or customization, these individuals at the lower end of the comfort limit range could also reach higher comfort limits. Regardless, every individual will have some force upper-bound imposed by comfort limits. For exosuit devices that are trying to maximize assistance, or which may be applying forces approaching the comfort limit for a given user, then slightly increasing the lever arm about biological joints provides one means of increasing torque assistance without increasing the pulling force [4], [5], [23]. Similar statements could be made about rigid exoskeletons and other wearable assistive devices as well: Regardless of whether the device is pushing, pulling, or compressing the user’s body segment there will be some comfort limit, and it is advantageous for device designers to have a rough range or benchmark data on what levels of force make users uncomfortable. This is one of the first studies to provide such benchmark data.

We found that, on average, the comfort limit increased by roughly 20–35% on each body segment over the four days of testing (Fig 3). These results suggest that, over time, users tolerated levels of force higher than their initial comfort limit on the first day; in fact, some participants mentioned they could perceive their tolerance going up over the course of the study. If a device provides assistance that is initially near a user’s comfort limit but below desired assistance levels, assistance could be increased over multiple days as the user habituates to the device. This finding also highlights the potential importance of multi-day training and device acclimation prior to assessing user comfort. Indeed, previous literature on footwear supports the idea that comfort perception often changes over time [18], and this is consistent with our own experiences and observations that wearable device users generally report increased levels of comfort over time as they habituate. It will be intriguing to explore whether, and to what extent, this comfort limit continues to increase over prolonged usage periods (e.g., weeks, months); but this was beyond this scope of this current study.

Higher rates of force application resulted in an average 5–20% increase in the comfort limit (Fig 4). Although statistical significance was mixed, in part due to the ceiling effect imposed by the maximum applied force threshold, this increase in comfort limit at higher rates appeared to occur for all three segments. This effect was seen despite the order of magnitude difference in the force rates between the shoulders (300 vs. 100 Ns^-1^) and the thigh/shank (3000 vs. 1000 Ns^-1^). This finding suggests that higher levels of exosuit assistance may be more tolerable during dynamic tasks that involve quick bursts of force, than during static postures that involve slower or sustained forces. Previous literature examining the effect of applied force rate using algometers also found that increasing rate of applied force increases comfort limits [22], [24]. One reason for this increase in comfort limit in our study may be that the force in the low loading rate trials was applied (and tissues strained) for a duration three times longer than that of the high rate trials.

This study focused on short-term comfort limits, using trials on the order of seconds. However, assistive exosuits are typically used, or designed to be used, on the order of minutes or hours. Our results assist with and enable longer-term study design, by providing some knowledge of and benchmark data on short-term comfort limits. The short-term comfort limit results place an upper-bound on what healthy end-users would likely tolerate in terms of applied forces. Short-term comfort limits may also be relevant to designing exosuits when assistance is only expected to be provided infrequently or intermittently, or for devices intended for short bouts (e.g., in emergency situations). More commonly, designers will likely want to design their devices to maintain forces well below the average comfort limits reported here, until longer-term studies or device- and task- specific data are available. Future experiments could explore the relationship between force magnitude and the length of time before a subject becomes uncomfortable, monitor how comfort limits change based on the duty cycle of force application, or create distribution graphs and/or prediction ellipses to estimate comfort limits given a confidence range. Due to time constraints we limited our testing to the shank, thigh, and shoulders in static postures. We explored exosuit interfaces, which pull axially along body segments, as opposed to exoskeleton interfaces that typically push orthogonally on body segments. However, even within the realm of exosuits there is variability in the angle of pull relative to each body segment, and it would be interesting to explore how comfort limits change as a function of angle. Future studies could use a similar protocol to the one detailed here to explore other segments of the body, or loading in other directions, which would be relevant to additional exosuits and exoskeletons. It would also be interesting to explore how a person’s comfort limit changes in different body postures, or during dynamic movement tasks. Since comfort is a subjective experience we could only ensure that everyone received the same instructions, but had no way to control how these were interpreted by each participant. Muscle fatigue is another possible confounding factor. However, the testing protocol each day was relatively short and there was no indication from subjects of muscle fatigue affecting comfort limits. In addition, subject data did not show evidence of the comfort limit lowering as the number of trials a subject or segment experienced increased. There are also multiple other factors that may affect comfort limits and which could be explored in future work, including gender, body mass index, pain tolerance, physical fitness and level of muscle activation. The results here provide novel benchmark data on comfort limits for healthy, relatively young adults; but future studies would be needed to characterize comfort limits for targeted patient populations and for older individuals.

## Conclusion

The comfort limits of the healthy adult participants in our study were generally on the order of about one body weight, but varied considerably between individuals, and increased across testing days. In addition, participants seemed to be more tolerant of higher force loading rates than lower ones. The results reported provide new and useful benchmarks for exosuit designers and end-users, and a scientific foundation for exploring comfort limits over larger time scales, in larger samples and in different populations.

## Supporting information

S1 Appendix Appendix contains data tables, additional figures on experimental setup, and specifications of the interfaces used. (DOCX)

S1 Data (MAT)
