# Supplementary material for: Characterizing the comfort limits of forces applied to the shoulders, thigh and shank to inform exosuit design
Source: PLoS One. 2020 Feb 12;15(2):e0228536. doi: 10.1371/journal.pone.0228536 (PMC7015417; doi:10.1371/journal.pone.0228536)
Supplement: S1 Appendix — (DOCX) [file pone.0228536.s001.docx]

**Appendix A: Data Tables.**

Summary of comfort limits across subjects, force rates, and days.

**Table A1:** Inter-subject comfort limit group means in Newtons (N). Comfort limit is max force experienced by a subject during a trial. See main text for a description of computational methods.

|  | | |
| --- | --- | --- |
| *N* = 10 | **Mean** | **Std** |
| Shoulders | 621 | 245 |
| Thigh | 867 | 296 |
| Shank | 702 | 220 |

**Table A2:** Inter-subject comfort limit metrics (N). Computed from all data from each subject. Subjects with an (*) only had two days of data.

|  |  | | | | | | |
| --- | --- | --- | --- | --- | --- | --- | --- |
|  | **Subject** | **Mean** | **Std** | **Range** | **Max** | **Min** | **Median** |
| Shoulders | **1** | 487 | 91 | 325 | 637 | 312 | 482 |
|  | **2** | 1105 | 123 | 390 | 1236 | 846 | 1181 |
|  | **3** | 723 | 150 | 553 | 992 | 439 | 720 |
|  | **4** | 538 | 107 | 402 | 739 | 336 | 546 |
|  | **5** | 399 | 74 | 346 | 617 | 271 | 390 |
|  | **6** | 648 | 93 | 421 | 912 | 492 | 640 |
|  | **7** | 257 | 34 | 122 | 328 | 206 | 260 |
|  | **8** | 704 | 118 | 484 | 963 | 479 | 702 |
|  | **9** | 653 | 173 | 605 | 943 | 338 | 669 |
|  | **10** | 629 | 105 | 374 | 839 | 465 | 619 |
|  | **Group (All Data)** | 614 | 242 | 1030 | 1236 | 206 | 597 |
|  |  |  |  |  |  |  |  |
|  | **Sub** | **Mean** | **Std** | **Range** | **Max** | **Min** | **Median** |
| Thigh | **1** | 838 | 224 | 807 | 1148 | 341 | 872 |
|  | **2** | 1125 | 54 | 171 | 1192 | 1022 | 1147 |
|  | **3** | 1057 | 146 | 543 | 1184 | 640 | 1129 |
|  | **4** | 900 | 207 | 674 | 1146 | 473 | 966 |
|  | **5** | 484 | 105 | 360 | 667 | 307 | 465 |
|  | **6** | 716 | 107 | 382 | 921 | 539 | 720 |
|  | **7** | 270 | 49 | 209 | 379 | 170 | 276 |
|  | **8** | 1020 | 121 | 449 | 1183 | 734 | 1036 |
|  | **9** | 894 | 224 | 664 | 1181 | 517 | 935 |
|  | **10** | 1075 | 136 | 541 | 1186 | 645 | 1123 |
|  | **Group (All Data)** | 838 | 302 | 1023 | 1192 | 170 | 922 |
|  |  |  |  |  |  |  |  |
|  | **Subject** | **Mean** | **Std** | **Range** | **Max** | **Min** | **Median** |
| Shank | **1** | 659 | 130 | 461 | 917 | 457 | 672 |
|  | **2** | 1085 | 87 | 320 | 1183 | 863 | 1126 |
|  | **3*** | 625 | 109 | 392 | 785 | 393 | 627 |
|  | **4*** | 552 | 111 | 389 | 805 | 415 | 539 |
|  | **5** | 506 | 99 | 569 | 934 | 365 | 513 |
|  | **6** | 735 | 130 | 551 | 962 | 410 | 731 |
|  | **7** | 371 | 61 | 290 | 541 | 251 | 365 |
|  | **8** | 852 | 128 | 522 | 1139 | 617 | 845 |
|  | **9*** | 656 | 76 | 291 | 856 | 565 | 672 |
|  | **10** | 927 | 142 | 513 | 1138 | 625 | 929 |
|  | **Group (All Data)** | 712 | 240 | 932 | 1183 | 251 | 693 |
|  |  |  |  |  |  |  |  |
|  |  |  |  |  |  |  |  |

**Table A3:** Inter-day comfort limit group means (N).

|  | | |
| --- | --- | --- |
| Shoulders | **Mean** | **Std** |
| Day 1 | 540 | 272 |
| Day 2 | 634 | 251 |
| Day 3 | 627 | 242 |
| Day 4 | 649 | 193 |
| Day Mean | **613** | ***N* = 10** |
|  |  |  |
| Thigh | **Mean** | **Std** |
| Day 1 | 709 | 288 |
| Day 2 | 837 | 293 |
| Day 3 | 873 | 300 |
| Day 4 | 954 | 307 |
| Day Mean | **843** | ***N* = 10** |
|  |  |  |
| Shank | **Mean** | **Std** |
| Day 1 | 658 | 200 |
| Day 2 | 708 | 264 |
| Day 3 | 740 | 262 |
| Day 4 | 806 | 299 |
| Day Mean | **728** | ***N* = 7** |

**Table A4:** Inter-day comfort limit metrics (N). Computed from all data for each day. Shoulder and thigh segment data is for *N* = 10 subjects; shank segment data is for *N* = 7 subjects.

|  |  | | | | | | |
| --- | --- | --- | --- | --- | --- | --- | --- |
|  | **Day** | **Mean** | **Std** | **Range** | **Max** | **Min** | **Median** |
| Shoulders | **1** | 547 | 265 | 1014 | 1222 | 207 | 491 |
|  | **2** | 630 | 248 | 1030 | 1236 | 206 | 622 |
|  | **3** | 632 | 235 | 995 | 1201 | 206 | 616 |
|  | **4** | 648 | 207 | 878 | 1145 | 266 | 638 |
|  |  |  |  |  |  |  |  |
| Thigh | **1** | 708 | 284 | 991 | 1192 | 202 | 693 |
|  | **2** | 824 | 283 | 980 | 1186 | 206 | 893 |
|  | **3** | 876 | 296 | 1012 | 1182 | 170 | 979 |
|  | **4** | 944 | 298 | 1007 | 1183 | 176 | 1117 |
|  |  |  |  |  |  |  |  |
| Shank | **1** | 676 | 219 | 841 | 1146 | 305 | 665 |
|  | **2** | 712 | 249 | 821 | 1130 | 309 | 722 |
|  | **3** | 746 | 248 | 874 | 1183 | 310 | 741 |
|  | **4** | 799 | 287 | 926 | 1177 | 251 | 867 |
|  |  |  |  |  |  |  |  |

**Table A5:** Force rate comfort limit group means (N).

| High | | |  | Low | | |
| --- | --- | --- | --- | --- | --- | --- |
| Shoulders | **Mean** | **Std** |  | **Shoulders** | **Mean** | **Std** |
| Day 1 | 603 | 272 |  | **Day 1** | 503 | 281 |
| Day 2 | 701 | 264 |  | **Day 2** | 559 | 231 |
| Day 3 | 692 | 256 |  | **Day 3** | 573 | 209 |
| Day 4 | 710 | 226 |  | **Day 4** | 580 | 179 |
| Day Mean | **677** | ***N* = 10** |  | **Day Mean** | **554** | ***N* = 10** |
|  |  |  |  |  |  |  |
| Thigh | **Mean** | **Std** |  | **Thigh** | **Mean** | **Std** |
| Day 1 | 733 | 306 |  | **Day 1** | 691 | 277 |
| Day 2 | 846 | 290 |  | **Day 2** | 828 | 297 |
| Day 3 | 906 | 322 |  | **Day 3** | 857 | 307 |
| Day 4 | 960 | 304 |  | **Day 4** | 946 | 324 |
| Day Mean | **861** | ***N* = 10** |  | **Day Mean** | **831** | ***N* = 10** |
|  |  |  |  |  |  |  |
| Shank | **Mean** | **Std** |  | **Shank** | **Mean** | **Std** |
| Day 1 | 726 | 259 |  | **Day 1** | 629 | 199 |
| Day 2 | 748 | 276 |  | **Day 2** | 684 | 244 |
| Day 3 | 775 | 258 |  | **Day 3** | 728 | 263 |
| Day 4 | 828 | 318 |  | **Day 4** | 768 | 295 |
| Day Mean | **769** | ***N* = 7** |  | **Day Mean** | **702** | ***N* = 7** |

**Table A6:** Force rate comfort limit metrics (N). Computed from all data for each day at each rate. Shoulder and thigh segment data is for *N* = 10 subjects; shank segment data is for *N* = 7 subjects.

|  |  | | | | | | |
| --- | --- | --- | --- | --- | --- | --- | --- |
| High Rate | **Day** | **Mean** | **Std** | **Range** | **Max** | **Min** | **Median** |
| Shoulders | **1** | 594 | 258 | 1003 | 1211 | 208 | 532 |
|  | **2** | 694 | 255 | 984 | 1205 | 221 | 727 |
|  | **3** | 691 | 247 | 957 | 1201 | 244 | 661 |
|  | **4** | 718 | 218 | 873 | 1145 | 272 | 687 |
|  |  |  |  |  |  |  |  |
| Thigh | **1** | 732 | 299 | 950 | 1152 | 202 | 706 |
|  | **2** | 835 | 280 | 941 | 1147 | 206 | 922 |
|  | **3** | 897 | 307 | 982 | 1152 | 170 | 1066 |
|  | **4** | 953 | 297 | 974 | 1150 | 176 | 1119 |
|  |  |  |  |  |  |  |  |
| Shank | **1** | 733 | 237 | 841 | 1146 | 305 | 758 |
|  | **2** | 744 | 261 | 821 | 1130 | 309 | 735 |
|  | **3** | 764 | 254 | 841 | 1150 | 310 | 827 |
|  | **4** | 827 | 300 | 897 | 1148 | 251 | 917 |
|  |  |  |  |  |  |  |  |
|  |  |  |  |  |  |  |  |
| Low Rate | **Day** | **Mean** | **Std** | **Range** | **Max** | **Min** | **Median** |
| Shoulders | **1** | 500 | 266 | 1014 | 1222 | 207 | 418 |
|  | **2** | 565 | 225 | 1030 | 1236 | 206 | 567 |
|  | **3** | 573 | 208 | 946 | 1152 | 206 | 553 |
|  | **4** | 579 | 170 | 686 | 952 | 266 | 572 |
|  |  |  |  |  |  |  |  |
| Thigh | **1** | 683 | 270 | 841 | 1192 | 250 | 656 |
|  | **2** | 812 | 288 | 821 | 1186 | 275 | 845 |
|  | **3** | 855 | 287 | 841 | 1182 | 277 | 937 |
|  | **4** | 936 | 302 | 897 | 1183 | 293 | 1081 |
|  |  |  |  |  |  |  |  |
| Shank | **1** | 620 | 186 | 575 | 939 | 365 | 625 |
|  | **2** | 681 | 237 | 719 | 1059 | 340 | 671 |
|  | **3** | 727 | 245 | 834 | 1183 | 349 | 719 |
|  | **4** | 771 | 275 | 847 | 1177 | 330 | 798 |
|  |  |  |  |  |  |  |  |

**Appendix B: Segment Orientations**

Subject orientations during testing in custom built scaffold.

**
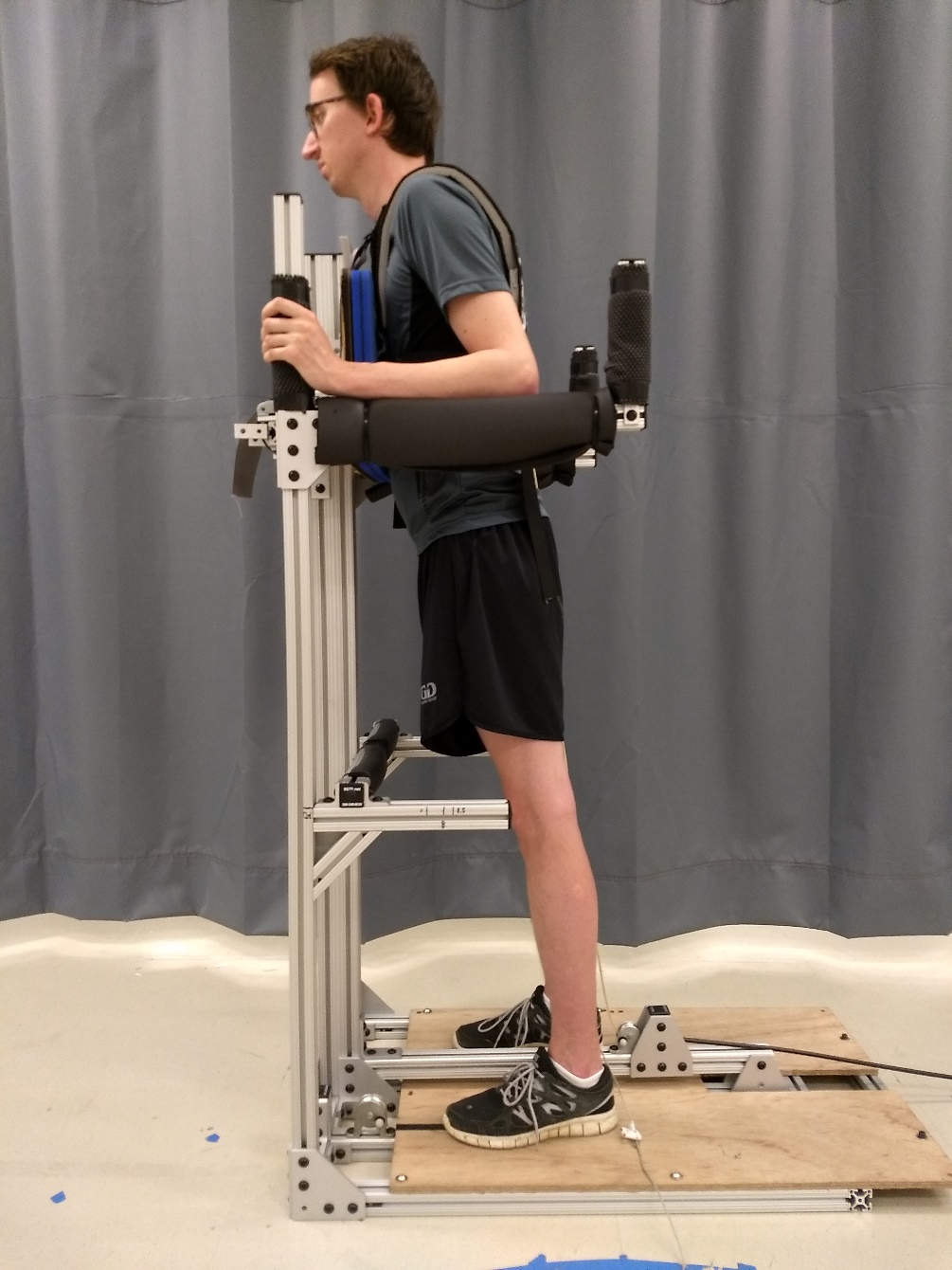

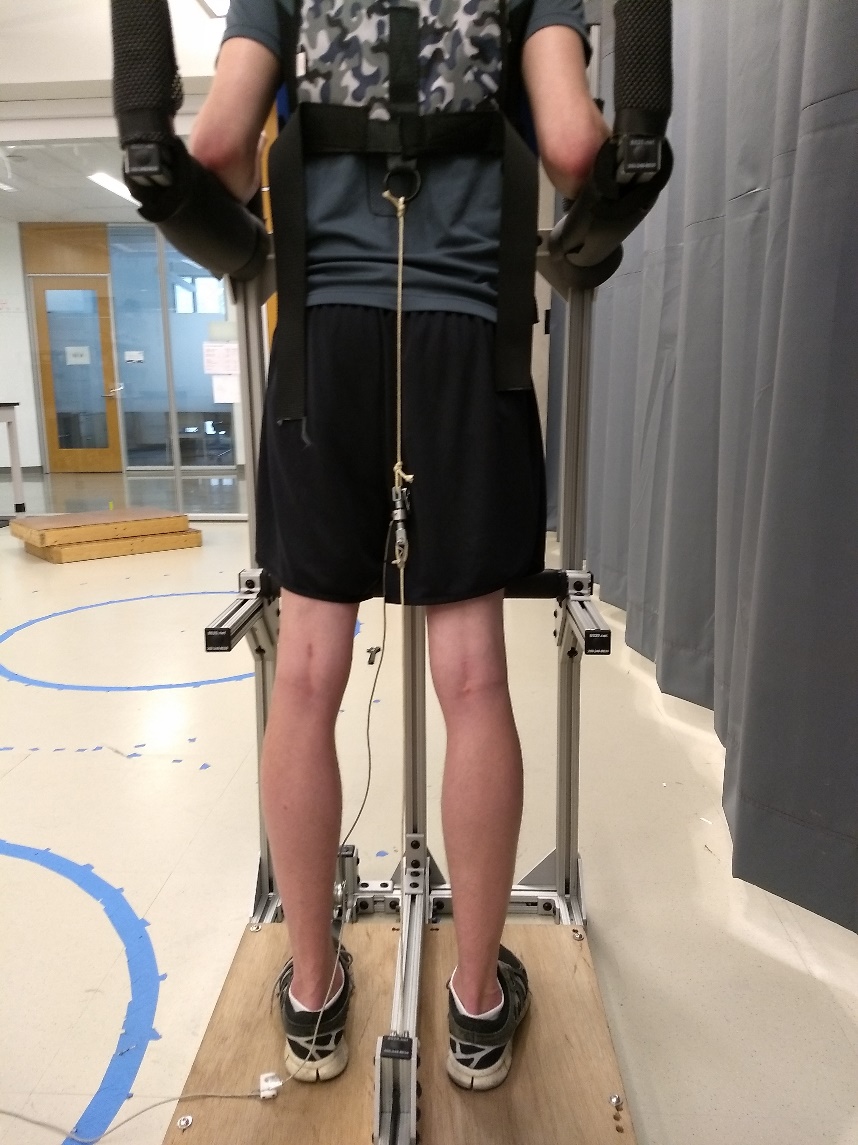
**

**Figure A1.** Subject orientation when testing the shoulder segment. The image above is not an original photo from the study, it is a reenactment photo used for illustrative purposes only.

**
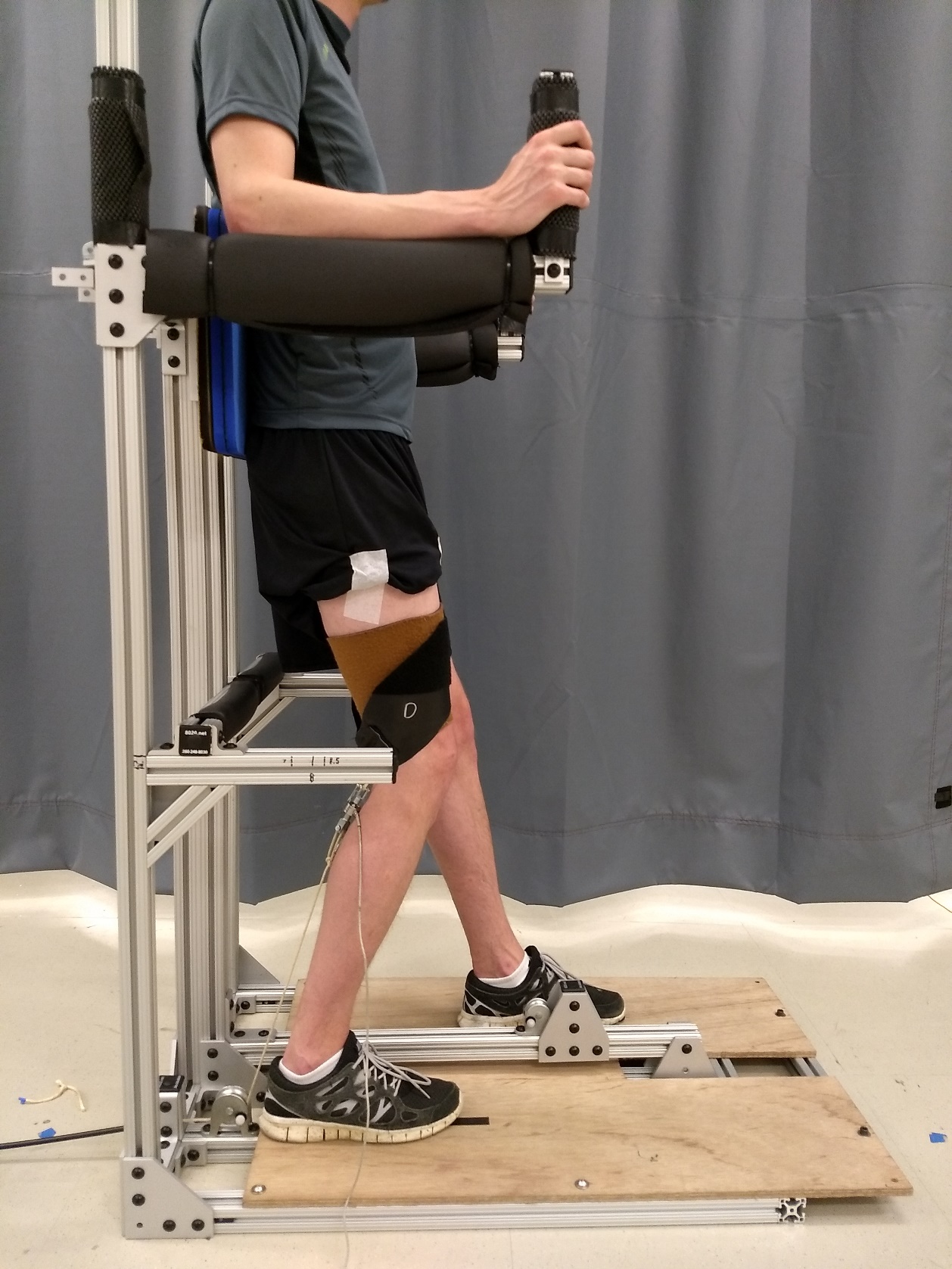
**

**Figure A2.** Subject orientation when testing the thigh segment. The image above is not an original photo from the study, it is a reenactment photo used for illustrative purposes only.

**
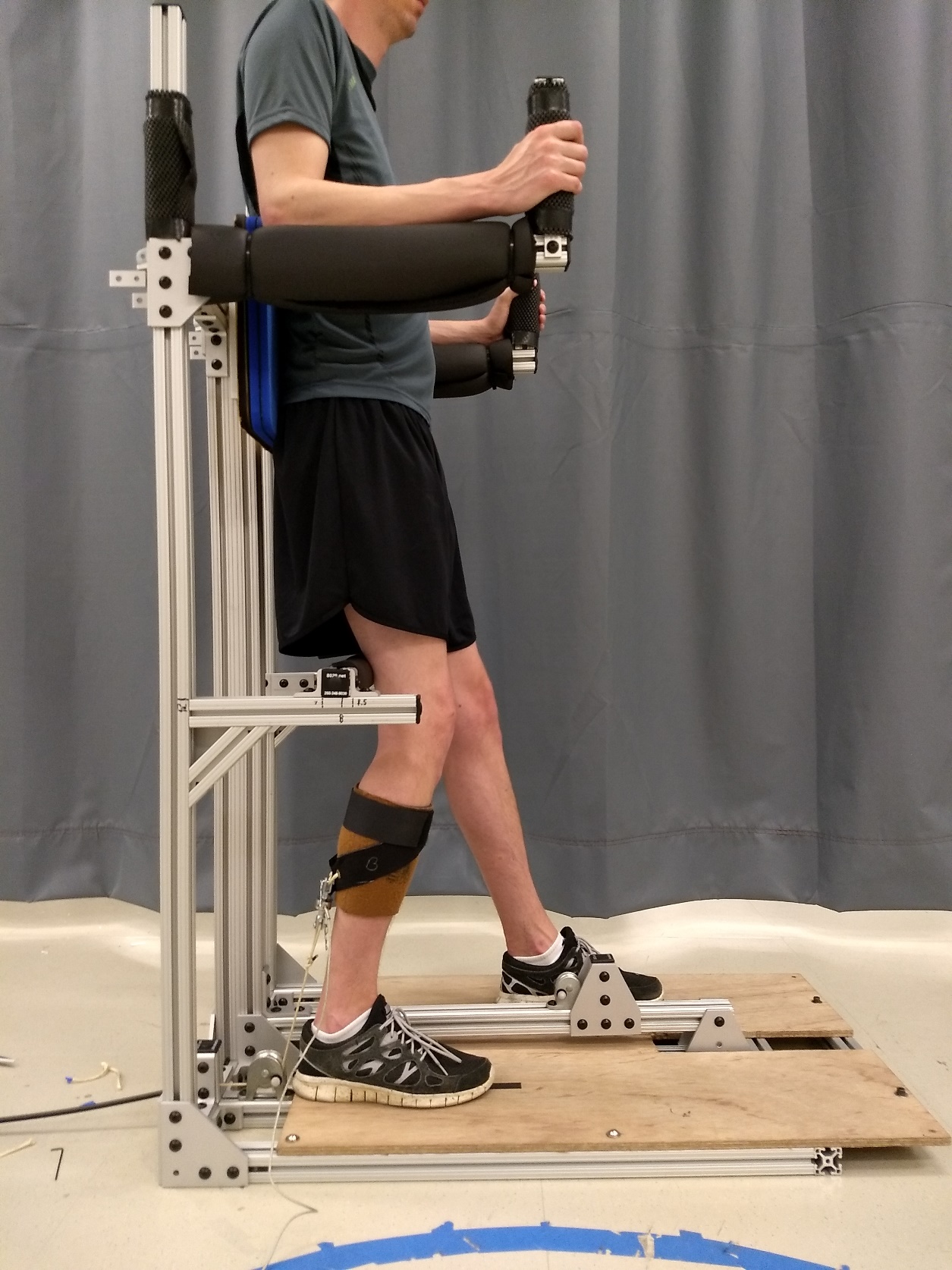
**

**Figure A3.** Subject orientation when testing the shank segment. The image above is not an original photo from the study, it is a reenactment photo used for illustrative purposes only.

**Appendix C: Interface Dimensions**

Dimensions of interfaces used in testing.


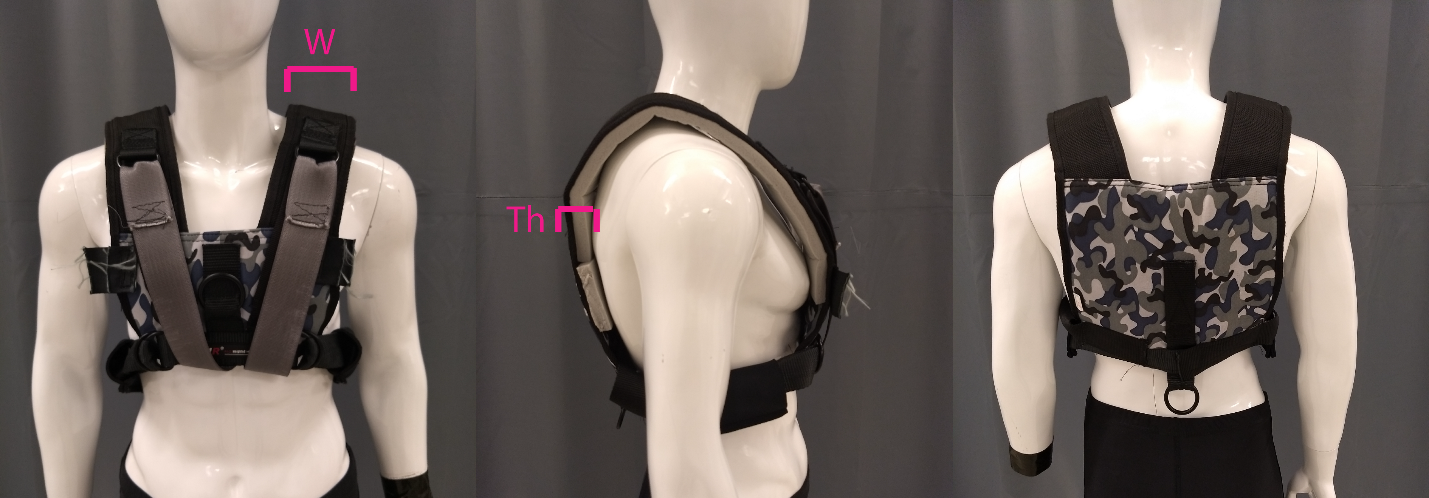


**Figure A4. Shoulder harness dimensions.** *W* = 76 mm, *Th* = 16 mm. Vinyl closed-cell foam was 76 mm wide x 13 mm thick (foam compresses 25% at 17.2 kPa, with a durometer < 30 on Shore 00 Scale).

**
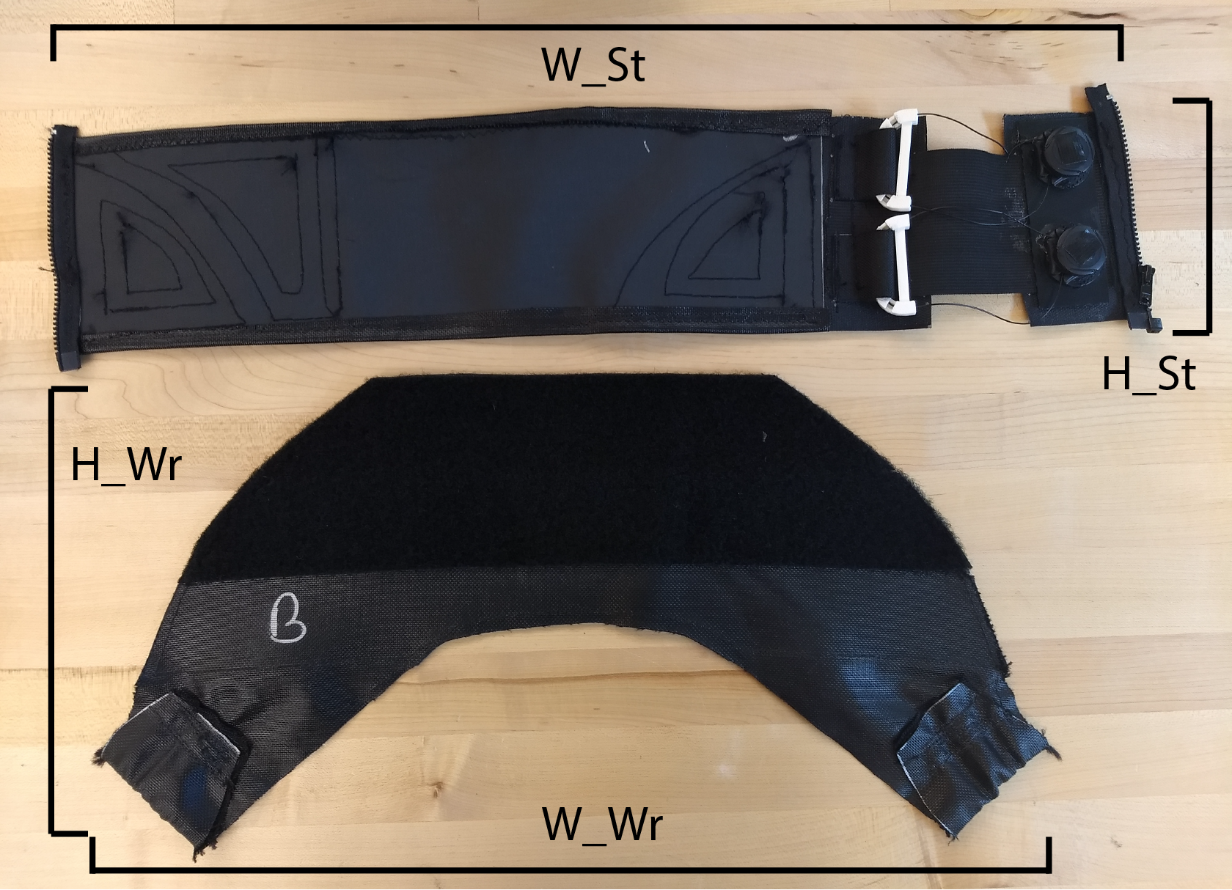
**

**Figure A5.** Thigh harness dimensions.

**Table A7:** Thigh wrap dimensions. Specifies height and width of thigh wrap (see Fig. A5).

|  |  | | | |
| --- | --- | --- | --- | --- |
| Size (mm) | **A** | **B** | **C** | **D** |
| H_Wr | 245 | 242 | 238 | 241 |
| W_Wr | 527 | 491 | 439 | 405 |

**Table A8:** Thigh strap dimensions. Specifies height and width of thigh strap (see Fig. A5).

|  |  | | | |
| --- | --- | --- | --- | --- |
| Size (mm) | **4** | **3** | **2** | **1** |
| H_St | 118 | 120 | 116 | 118 |
| W_St | 632 | 588 | 540 | 489 |

**
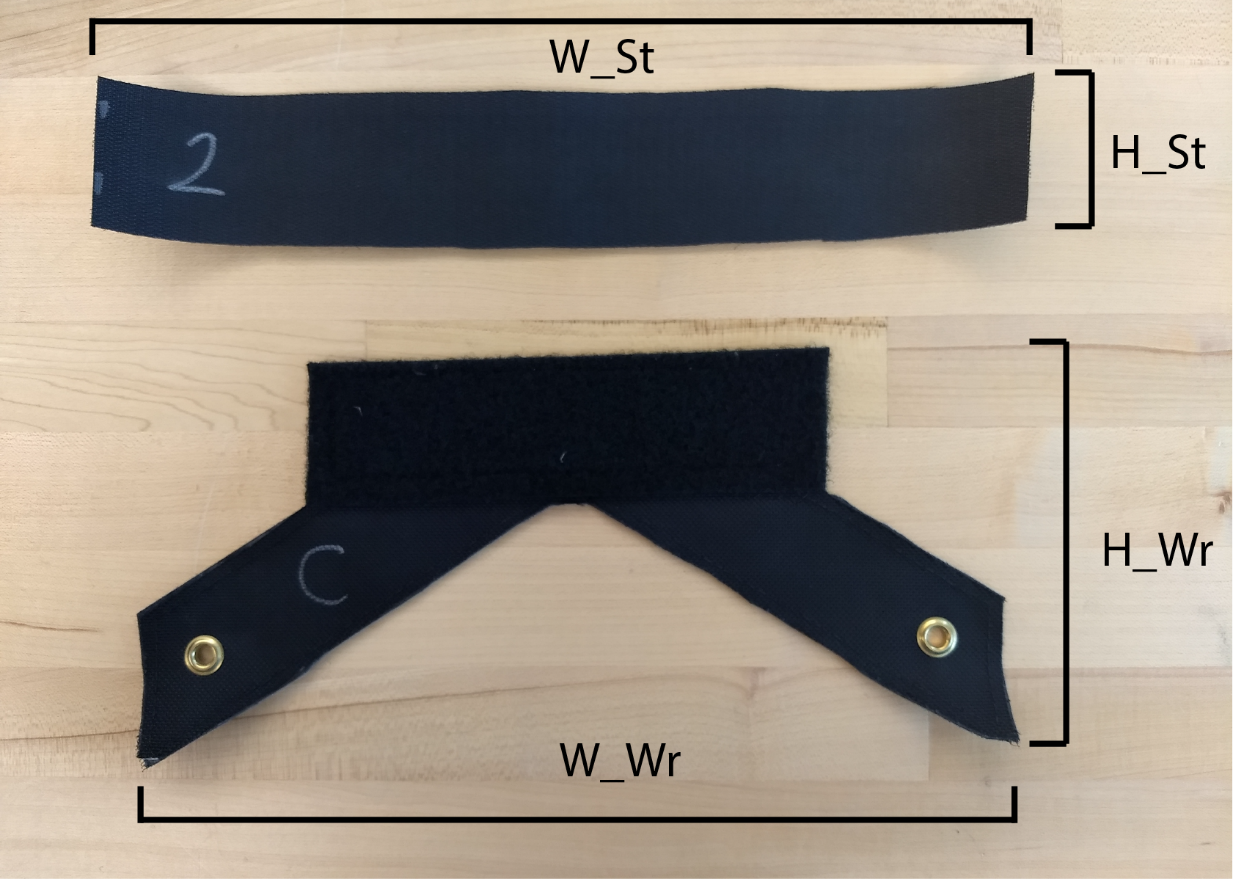
**

**Figure A6.** Shank harness dimensions.

**Table A9:** Shank wrap dimensions. Specifies height and width of shank wrap (see Fig. A6).

|  | Shank Wrap | | | |
| --- | --- | --- | --- | --- |
| Size (mm) | **A** | **B** | **C** | **D** |
| H_Wr | 182 | 169 | 152 | 125 |
| W_Wr | 406 | 357 | 311 | 233 |

**Table A10:** Shank strap dimensions. Specifies height and width of shank strap (see Fig. A6).

|  | Shank Strap | | |
| --- | --- | --- | --- |
| Size (mm) | 1 | 2 | 3 |
| H_St | 53.5 | 55 | 52 |
| W_St | 394 | 327 | 266 |

**
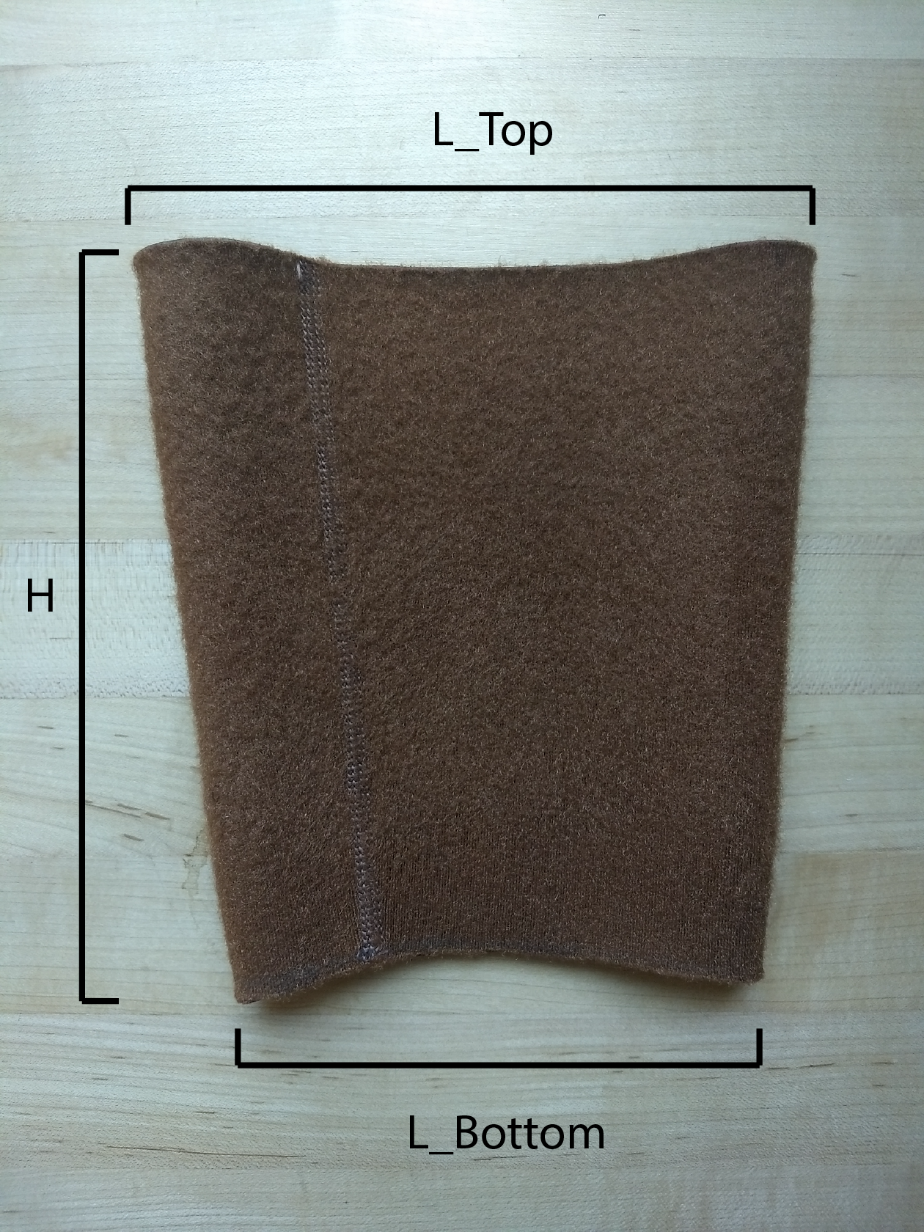
**

**Figure A6:** Thigh and shank sleeve dimensions. Liner thickness was about 4 mm.

**Table A11:** Thigh and shank sleeve dimensions.

|  | Thigh Sleeve | | |  | Shank Sleeve | | |
| --- | --- | --- | --- | --- | --- | --- | --- |
|  | **Large** | **Medium+** | **Medium** |  | **Large** | **Medium+** | **Medium** |
| L_Top (mm) | 209 | 195 | 130 |  | 175 | 158 | 124 |
| L_Bottom (mm) | 174 | 156 | 121 |  | 145 | 122 | 113 |
| H (mm) | 214 | 205 | 203 |  | 179 | 176 | 179 |
| Circumference (Top, mm) | 418 | 390 | 261 |  | 349 | 315 | 249 |
| Circumference (Bottom, mm) | 349 | 312 | 242 |  | 289 | 244 | 226 |
| Surface Area (mm^2^) | 40846 | 35754 | 25529 |  | 28511 | 24488 | 21230 |
